# Impact of Clinical and Morphological Factors on Long-Term Mortality in Patients with Myocardial Bridge

**DOI:** 10.3390/jcdd9050129

**Published:** 2022-04-25

**Authors:** György Bárczi, Dávid Becker, Nóra Sydó, Zoltán Ruzsa, Hajnalka Vágó, Attila Oláh, Béla Merkely

**Affiliations:** 1 Heart and Vascular Center, Semmelweis University, 1122 Budapest, Hungary; barczigyorgy@gmail.com (G.B.); becker.david@kardio.sote.hu (D.B.); nora.sydo@gmail.com (N.S.); zruzsa25@gmail.com (Z.R.); vagoha@gmail.com (H.V.); merkely.study@gmail.com (B.M.); 2 2nd Department of Internal Medicine, Division of Invasive Cardiology, University of Szeged, 6720 Szeged, Hungary

**Keywords:** angina pectoris, bridge morphology, myocardial bridging, quantitative coronary analysis, survival analysis

## Abstract

Although myocardial bridging (MB) has been intensively investigated using different methods, the effect of bridge morphology on long-term outcome is still doubtful. We aimed at describing the anatomical differences in coronary angiography between symptomatic and non-symptomatic LAD myocardial bridges and to investigate the influence of clinical and morphological factors on long-term mortality. In our retrospective, long-term, single center study we found relevant MB on the left anterior descendent (LAD) coronary artery in 146 cases during a two-year period, when 11,385 patients underwent coronary angiography due to angina pectoris. Patients were divided into two groups: those with myocardial bridge only (LAD-MB^neg^, *n* = 78) and those with associated obstructive coronary artery disease (LAD-MB^pos^, *n* = 68). Clinical factors, morphology of bridge by quantitative coronary analysis and ten-year long mortality data were collected. The LAD-MB^neg^ group was associated with younger age and decreased incidence of diabetes mellitus, as well as with increased minimal diameter to reference diameter ratio (LAD-MB^neg^ 54.5 (13.1)% vs. LAD-MB^pos^ 46.5 (16.4)%, *p* = 0.016), while there was a tendency towards longer lesions and higher vessel diameter values compared to the LAD-MB^pos^ group. The LAD-MB^pos^ group was associated with increased mortality compared to the LAD-MB^neg^ group. The analysis of our data showed that morphological parameters of LAD bridge did not influence long-term mortality, either in the overall population or in the LAD-MB^neg^ patients. Morphological parameters of LAD bridge did not influence long-term mortality outcomes; therefore, it suggests that anatomical differences might not predict long-term outcomes and should not influence therapy.

## 1. Introduction

Myocardial bridging (MB) is a relatively common congenital coronary anomaly, in which a segment of coronary artery takes an intramuscular course that causes vessel compression during systole [1]. The prevalence of MB is highly variable and depends on the investigational method: its frequency has been described as 1.5 to 16% when assessed by angiography [2]; however, in an autopsy series it has been shown to be as high as 80% [3], while it was 44% in a study using 64-slice multidetector computed tomographic angiography [4]. Although MB can be found in any epicardial artery, it is most frequently localized to the mid-segment of the left anterior descendent artery (LAD) on coronary angiography [5].

While myocardial bridging is generally considered as a benign phenomenon and related serious events are uncommon, many cases have been reported where MB was considered responsible for a wide variety of cardiovascular symptoms and complications, including angina, acute coronary syndrome, left ventricular dysfunction, arrhythmias and even sudden cardiac death [5,6,7,8,9,10]. By using novel invasive methods (fractional flow reserve, intracoronary Doppler and intravascular ultrasound), our understanding about the pathophysiology has been expanded [11,12,13,14,15]. Not only the compression during systole, but also the accelerated atherosclerosis proximal to the bridged segment, permanent reduction in the diastolic diameter and impaired flow reserve contribute to the relative ischemia [1].

Despite these data, the prognostic factors and the clinical evaluation of MB has remained limited. Moreover, the relationship between anatomy and potentially dangerous MBs has not yet been clarified. In this retrospective, high-number population-based clinical study we aimed at describing the anatomical differences between symptomatic and non-symptomatic LAD myocardial bridges found on coronary angiography and to investigate the influence of clinical and bridge morphological factors on long-term mortality.

## 2. Materials and Methods

A total of 11,385 diagnostic coronary angiographies were performed (indications according to the current European guidelines) in our cardiology center, at the Heart and Vascular Center of the Semmelweis University of Budapest between 25 March 2009 and 12 March 2011. Ethical approval (of enrolled patients) was obtained from the Central Ethics Committee of Hungary, with all of the participants completing informed consent forms, which were in conformity with the WMA Declaration of Helsinki-Ethical Principles for Medical Research Involving Human Subjects.

**Figure 1:**
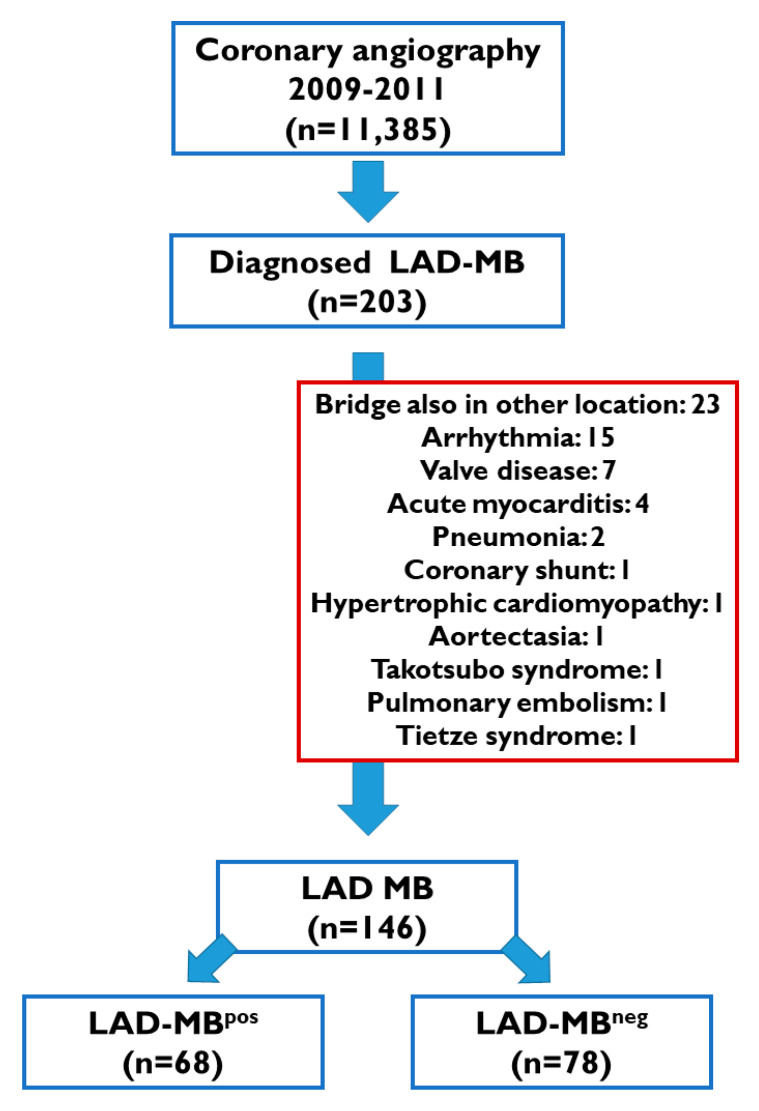
The design of the study and the selection of patients. LAD-MB: Myocardial bridge of left anterior descendent coronary artery; LAD-MB^pos^: Left anterior descendent myocardial bridge with another significant atherosclerotic coronary lesion group; LAD-MB^neg^: Left anterior descendent myocardial bridge without another significant atherosclerotic coronary lesion group.

We enrolled a total of 203 patients (1.78%) with a clear presence of MBs (defined as a visible alteration of vessel caliber between systole and diastole, recognized by the investigator). To obtain a more consistent study group, we excluded patients with MBs affecting arteries other than the LAD and where any other obvious cause of angina pectoris was presented (*n* = 57), see Figure 1. The remaining patients (*n* = 146) were divided into two groups according to accompanying coronary artery disease. Seventy-eight patients were referred for angiography because of typical angina pectoris, and, except for LAD MB, no other underlying, epicardial coronary disease was found (LAD-MB^neg^ group). Sixty-eight patients were also referred for angiography because of angina pectoris, but in this group significant coronary artery disease was also revealed in addition to the LAD MB (LAD-MB^pos^ group). In the LAD-MB^pos^ group coronary plaque was considered significant if lumen narrowing (>50% in diameter) of significant epicardial coronary arteries (>1.5 mm diameter) was observed, and we provided therapy according to current guidelines. Ad-hoc percutaneous coronary intervention was performed in 23 (34%) of these patients, with indications according to current guidelines.

For a better understanding, the design of the study and the selection of the patients are shown in Figure 1.

**Figure 2:**
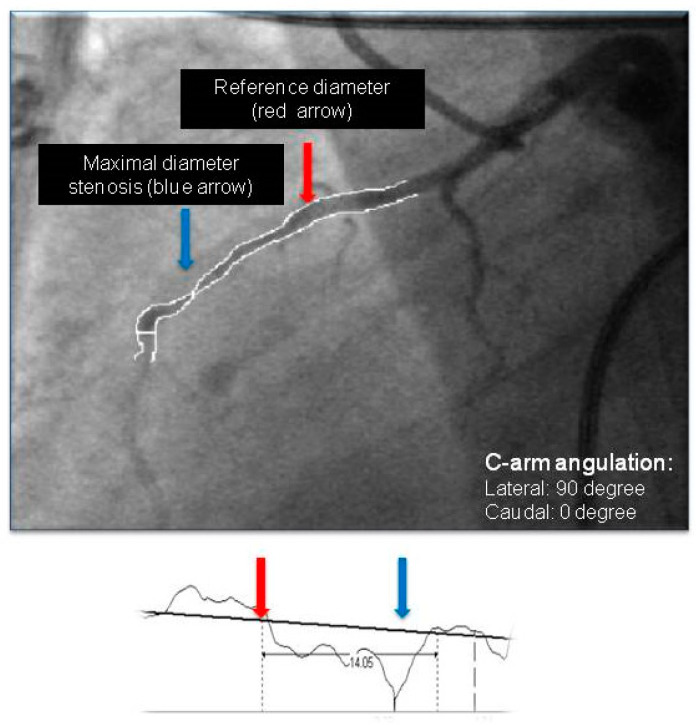
Representative image of a measurement process in lateral view (end-systole) by quantitative coronary angiography (QCA).

Quantitative angiography was performed according to our standard clinical practice. Vessels and lesions were analyzed using a computerized quantification system (Innova 2100, General Electric Medical Systems, Milwaukee, WI, USA). Measurements were obtained with digital calipers. All of the MBs were measured in lateral view (angulation of the “C” arm: left lateral, LAO: 90 degrees, caudal: 0 degrees) in end-systole and end-diastole by an expert interventionist (Figure 2). Three main parameters were measured: (1) length of the MB, defined as the distance from the most proximal point to the most distal point of the LAD, where the systolic narrowing phenomenon could be observed; (2) reference diameter of the MB, defined as the diameter of the vessel immediately proximal to the point where the systolic narrowing started; and (3) minimal diameter of the MB, measured also in end-systole at the point where the thickening was the most prominent. Additionally, from these parameters we calculated (4) minimal diameter to reference diameter, the ratio between minimal stenosis and reference diameter in percentage to characterize shortening of bridge for each patient.

In addition to these data, we also recorded height, weight, sex of patients and presence of main cardiovascular risk factors, such as diabetes mellitus, hypertension, and dyslipidemia.

We collected information about the patients’ mortality by phone visit and we also checked data on survival status (according to the Hungarian National Database) on 1 April 2020.

### Statistics

GraphPad Prism (version 6, GraphPad Software, San Diego, CA, USA) and SPSS (version 22, SPSS Inc., Chicago, IL, USA) were used for the statistical analysis.

We compared the LAD-MB^neg^ and LAD-MB^pos^ groups directly. In the case of normal distribution of continuous data, the unpaired *t*-test was used. In the case of non-normal distribution, the Mann–Whitney U-test was utilized. To compare parameters with binomial outcomes (sex, hypertension, hyperlipidemia and diabetes mellitus), the Chi-square test was performed.

Mortality rate was summarized by constructing Kaplan–Meier curves, and the distributions of the groups were compared by a log-rank test. For this analysis, median values were used to dichotomize continuous variables (MB length, reference diameter, minimal diameter and minimal diameter to reference diameter).

Single variable Cox regression analysis was used for the search of predictors of death from the data of the patients (age, sex, BMI, hypertension, hyperlipidemia and diabetes mellitus). All variables associated with a *p* value < 0.15 by single variable analysis were entered into the multiple variable Cox regression analysis with the parameters of myocardial bridge (MB length, reference diameter, minimal diameter and minimal diameter to reference diameter). Hazard ratio was given with 95% CI interval and *p* value was provided for the significance of different parameters on clinical outcomes.

Continuous data are expressed as means with standard deviation.

A *p* value < 0.05 was considered significant for all tests.

## 3. Results

Demographic and clinical data and LAD-MB morphological data are summarized in Table 1. The total number of analyzed patients with LAD-MB was 146, and 64% were male with no differences between the LAD-MB^pos^ and LAD-MB^neg^ groups regarding sex. The LAD-MB^pos^ patients were characterized by older age and increased presence of type 2 diabetes mellitus, while there was no statistical difference between the presence of hypertension or hyperlipidemia and BMI.

MB morphological data are also presented in Table 1. According to our results, the LAD-MB^neg^ group was characterized with more severe morphological features. The shortening of MB (minimal diameter to reference diameter) significantly decreased, while the length and reference diameter showed a strong tendency towards the decreased value in the LAD-MB^neg^ group compared to the LAD-MB^pos^ group. The minimal diameter showed no differences between our groups.

**Figure 3:**
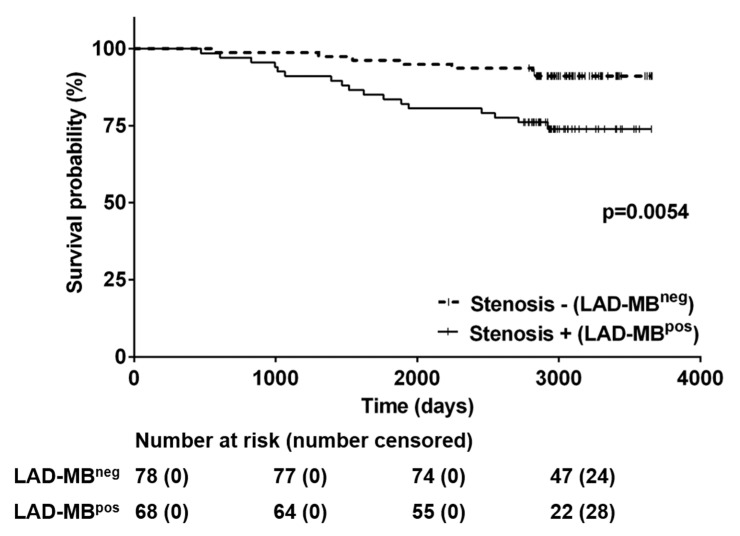
Kaplan–Meier curve of the long-term follow-up comparing LAD-MB^pos^ (left anterior descendent myocardial bridge with another significant atherosclerotic coronary lesion) and LAD-MB^neg^ (left anterior descendent myocardial bridge without another significant atherosclerotic coronary lesion) group. The LAD-MB^pos^ group was associated with higher mortality.

Long term follow-up: The average follow-up period of this patient population was 3115 (249) days, almost ten years. When we checked survival status, we found that mortality was 16.4% in the overall LAD-MB population. Eight people died in the LAD-MB^neg^ group; thus, the all-cause mortality rate in this population with isolated LAD-MB was 10.3% for this follow-up period. We searched for differences between the two prespecified subgroups: the Kaplan–Meier analysis revealed significant disparity in mortality between the LAD-MB^neg^ and LAD-MB^pos^ groups (Figure 3).

**Figure 4:**
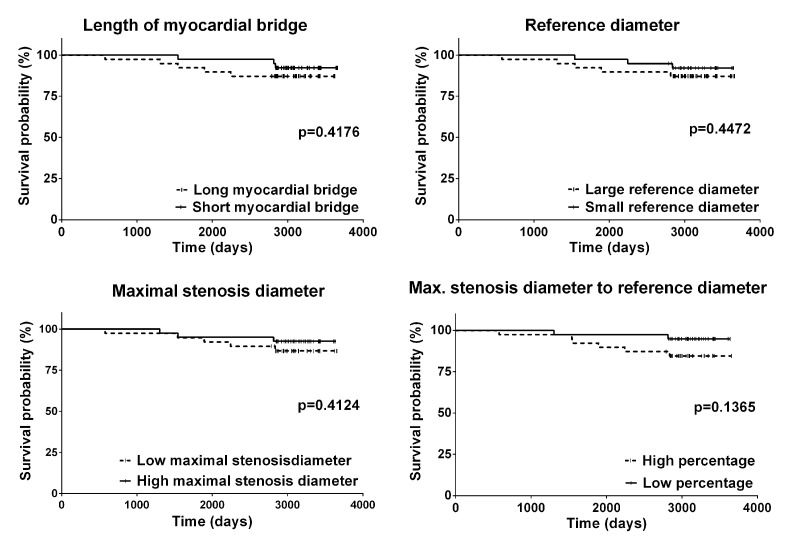
Kaplan–Meier curve of the long-term follow-up comparing morphological features of the myocardial bridge in LAD-MB^neg^ (left anterior descendent myocardial bridge without another significant atherosclerotic coronary lesion) group. For this analysis, median values were used to dichotomize continuous variables. None of the morphological characteristics influenced the mortality rate.

We also searched for factors that could influence long-term mortality both in overall LAD-MB population and in LAD-MB^neg^ group. As a first step, we determined factors from the demographic and clinical data using single variable Cox regression (Table 2). In the overall LAD-MB population the presence of coronary stenosis and diabetes mellitus and older age were associated with increased mortality, while in the LAD-MB^neg^ group only age influenced survival outcome. To determine the role of morphological parameters, a multiple variable Cox regression was performed with the morphological parameters (MB length, reference diameter, minimal diameter, minimal diameter to reference diameter) and influencing factors from single variable analysis. Our results show that none of the morphological parameters influence mortality (Table 2). This result was also confirmed by Kaplan–Meier analysis, when median values were used to dichotomize variables of MB morphology (Figure 4).

## 4. Discussion

Here we provided a retrospective, single-center study using long-term follow-up, with a moderate number of patients, to investigate anatomical symptomatic LAD myocardial bridges and to determine the role of clinical, demographic and morphological features in the long-term survival of the patients with LAD MB.

Although myocardial bridging has been intensively investigated since it was recognized by autopsy and coronary angiography, the exact potential effect on cardiovascular mortality is still unknown. Coronary angiography remained the gold standard for detecting MB; however, it has recently been more frequently revealed by MDCT (3.5–58%) than by angiography (0.4–15.8%) [16,17,18]. Angiography detects bridging at rates from 0.5% to 12%, most frequently localized on the middle segment of the LAD artery. The prevalence of LAD-MBs (1–2%) in our population was similar compared to the findings in other large-volume centers, detected by routine coronary angiography (Figure 1) [2].

The morphological parameters of these bridges, measured by quantitative coronary analysis (both the length and reference diameter values), are also comparable with the data from literature (Table 1) [1,19,20]. We used QCA, that gave us the opportunity to measure simple parameters describing the LAD bridge and might be utilized routinely and promptly during invasive measurements (Figure 2). There are more sophisticated invasive methods to characterize a bridge in detail; however, they need temporal and material sacrifice. Invasive imaging and functional assessment of the severity of MBs is possible with angiography, intravascular ultrasound (IVUS), optical coherence tomography (OCT) and fractional flow reserve (FFR) measurement [13,14,15]. IVUS demonstrated characteristic systolic compression of the bridge segments (the so-called half-moon phenomenon) and atherosclerosis, mostly predominant in the proximal segment [21]. Recently, there have been some reports of the usefulness of OCT in the evaluation of the internal coronary artery wall of myocardial bridges and MBs investigated by OCT, and they were found to be longer, but the diameter stenosis was lower than with angiography-based measurements [14]. The use of fractional flow reserve (FFR) is controversial because MB is a dynamic stenosis, and FFR has not been validated in MB; however, some reports exist of FFR-guided coronary intervention in MB [15].

Clinical adjudication of myocardial bridges is often difficult. On the one hand, there is an essential discrepancy between the high prevalence of the phenomenon accompanied with excellent prognosis (which was also observed in this study) [22,23,24,25] and between the numerous case reports describing serious clinical significance to tunneled arteries, mostly without detailed anatomical descriptions of these MBs. On the other hand, the proof of functional significance–linkage with clinical symptoms–is challenging with either invasive or non-invasive tests in particular. Large, controlled clinical trials are very limited in this field mainly because of the suspected benign nature of this unique coronary anomaly, moreover the model of MB has not been successfully established in experimental models either. It would be essential to clarify which tunneled arteries are potentially symptomatic and which are potentially life-threatening, and this might even cause sudden cardiac death.

We divided our population with angina pectoris, where obstructive coronary disease (>50% coronary stenosis) was also present, to examine whether in this population–where myocardial bridge might be only an accidental finding–there are different clinical factors and bridge features compared to patients with isolated LAD MB (Table 1). The cut-off of 50% diameter stenosis on epicardial vessels for defining obstructive CAD is based on studies on what degree of stenosis is flow limiting and may cause ischemia under stress [26]. It is not surprising that the LAD-MB^pos^ patients were older and could be characterized by an increased prevalence of type 2 diabetes mellitus and were associated with higher mortality compared to the LAD-MB^neg^ group (Figure 3). From another view, age and diabetes mellitus were the only risk factors that showed significant differences in the prevalence between the two groups. The high prevalence of hypertension (Table 1)–despite much younger patients in the LAD-MB^neg^ group–suggests that elevated arterial pressure might play an important role in the development of symptoms in the case of LAD-bridge, which was not observed in previous studies [27]. Symptomatic, isolated LAD-bridges were associated with more severe lumen narrowing, longer bridge segments and increased reference diameter (Table 1). The first-line treatment for symptomatic MB is medical therapy with beta and calcium channel blockers, which was provided to our patients [5]. The placement of metal stents in MB might be associated with stent fracture and edge restenosis; therefore, it should only be considered in patients with bridging refractory to medical therapy [28].

Isolated myocardial bridging is generally considered as a benign condition. Beside appealing, unique clinical cases [6,7,8,9], studies with long-term follow-up suggest that bridging might be associated with negative clinical outcomes, such as myocardial infarctions and arrhythmias [29]. In particular, long bridges were associated with these complications. The 10.2% 10-year long mortality in the LAD-MB^neg^ group is comparable with 1.1% average yearly mortality in 45–59 years old men and women in Hungary (WHO statistics, 2014). Therefore, we investigated in the LAD-MB^neg^ group whether the anatomical parameters measured by QCA might influence long-term mortality (Figure 4). We found that patients in the isolated myocardial bridging group were associated with low mortality and none of the anatomical factors or morphological severity influenced long-term (a follow-up of approximately 10 years) mortality (Figure 4, Table 2). Our data also suggest that more severe bridge anatomical characteristics are not associated with worse long-term outcomes, suggesting the benign nature of myocardial bridges independently from the severity of anatomical features.

### Limitations of the Study

The main limitations of the study were the retrospective design of the study and the lack of non-invasive assessment of MB anatomy with MDCT and invasive imaging techniques, such as IVUS or OCT. Although these measurements, which are not routinely used, would be more precise to describe bridge morphology, they require more financial and medical effort and are associated with increased radiation time. We also emphasize that no healthy control group was investigated to compare mortality directly to these individuals.

## 5. Conclusions

Here we provided a retrospective, single-center study using long-term follow-up. Morphological parameters of angina-associated, isolated LAD bridges were more severe compared to bridges that were accidentally found in patients with obstructive coronary disease. Our ten-year long follow-up period showed that morphological parameters measured with QCA did not influence long-term mortality outcomes; therefore, it suggests that anatomical differences might not predict long-term outcomes. Our data underline the benign nature of myocardial bridge.

## Figures and Tables

**Table 1:** The distribution of the data of patients presenting with angina pectoris and with a myocardial bridge detected in the left anterior descendent artery (*n* = 146) and comparison of the LAD-MB^neg^ and LAD-MB^pos^ population.

	Overall LAD-MB Population *n* = 146	LAD-MB^neg^ *n* = 78	LAD-MB^pos^ *n* = 68	LAD-MB^neg^ vs. LAD-MB^pos^
Mean age (years)	60.6 (12.7)	57.6 (12.4)	64.5 (11.5)	0.001
Male sex	94 (64%)	50 (64%)	43 (64%)	0.99
Hypertension	105 (72%)	57 (73%)	48 (72%)	0.87
Type 2 diabetes mellitus	36 (25%)	13 (17%)	24 (36%)	0.008
Hyperlipidemia	77 (53%)	37 (47%)	40 (60%)	0.14
Body mass index (kg/m^2^)	27.6 (3.8)	27.2 (3.4)	28.2 (4.3)	0.11
LAD-MB length (mm)	21.4 (8.2)	23.4 (8.3)	20.0 (7.7)	0.05
Reference diameter (mm)	2.18 (0.46)	2.23 (0.42)	2.09 (0.41)	0.06
Minimal diameter (mm)	1.10 (0.41)	1.02 (0.36)	1.11 (0.38)	0.39
Minimal diameter to reference diameter (%)	49.5 (15.5)	54.5 (13.1)	46.5 (16.4)	0.006

Data is shown as mean (SD). LAD-MB: Left anterior descendent myocardial bridge; LAD-MB^pos^: Left anterior descendent myocardial bridge with another significant atherosclerotic coronary lesion; LAD-MB^neg^: Left anterior descendent myocardial bridge without another significant atherosclerotic coronary lesion.

**Table 2:** Summary of univariate and multivariate Cox regression analysis of overall survival in overall population and LAD-MB^neg^ group.

	Overall LAD-MB Population *n* = 146		LAD-MB^neg^ *n* = 78	
	Single Variable Analysis	Multiple Variable Analysis	Single Variable Analysis	Multiple Variable Analysis
Stenosis	HR: 3.45 *p* = 0.005	HR: 2.14 (0.84–5.46) *p* = 0.111	NA	NA
Mean age (years)	HR: 1.08 (1.04–1.12) *p* < 0.001	HR: 1.08 (1.03–1.13) *p* = 0.001	HR: 1.07 (1.01–1.13) *p* = 0.033	HR: 1.09 (1.01–1.18) *p* = 0.030
Male sex	HR: 0.89 (0.40–1.98) *p* = 0.770	NA	HR: 0.56 (0.14–2.26) *p* = 0.419	NA
Hypertension	HR: 1.26 (0.50–3.17) *p* = 0.618	NA	HR: 1.07 (0.22–5.30) *p* = 0.934	NA
Type 2 diabetes mellitus	HR: 2.14 (0.96–4.77) *p* = 0.062	HR: 1.62 (0.71-3.71) p=0.251	HR: 0.69 (0.09–5.59) *p* = 0.726	NA
Hyperlipidaemia	HR: 0.69 (0.31–1.52) *p* = 0.355	NA	HR: 0.64 (0.15–2.69) *p* = 0.546	NA
BMI (kg/m^2^)	HR: 1.01 (0.91–1.12) *p* = 0.857	NA	HR: 1.01 (0.82–1.24) *p* = 0.954	NA
LAD-MB length (mm)	NA	HR: 1.01 (0.96–1.06) *p* = 0.827	NA	HR: 1.07 (0.97–1.19) *p* = 0.150
Reference diameter (mm)	NA	HR: 2.73 (0.17–44.8) *p* = 0.482	NA	HR: 6.30 (0.03–1534)] *p* = 0.512
Minimal stenosis (mm)	NA	HR: 0.45 (0.01–86.8) *p* = 0.768	NA	HR: 0.02 (0.01–1568) *p* = 0.503
Minimal stenosis to reference diameter (%)	NA	HR: 0.98 (0.88–1.09) *p* = 0.704	NA	HR: 0.94 (0.74–1.19) *p* = 0.592

LAD-MB: Left anterior descendent myocardial bridge; LAD-MB^neg^: Left anterior descendent myocardial bridge without another significant atherosclerotic coronary lesion group. HR: hazard ratio (95% CI for HR).

## Data Availability

The data presented in this study are available on request from the corresponding author.
